# Maternal vitiligo is associated with increased autoimmune and atopic disease in offspring: A nationwide birth cohort study

**DOI:** 10.1016/j.jdin.2025.12.011

**Published:** 2026-01-06

**Authors:** Hyun Jung Kim, Hei Sung Kim

**Affiliations:** a Department of Preventive Medicine, College of Medicine, Korea University, Seoul, Republic of Korea; b Department of Dermatology, Incheon St. Mary’s Hospital, The Catholic University of Korea, Seoul, Republic of Korea

**Keywords:** atopic disease, autoimmune disease, long-term outcomes, maternal vitiligo, neuropsychiatric disorders, offspring

*To the Editor*: Vitiligo is an autoimmune depigmenting disorder associated with other autoimmune conditions through shared genetic and immunologic pathways.^1^ Although familial clustering is well documented,^2^ the long-term health consequences for children of mothers with vitiligo remain unclear. Using nationwide linked data, we examined whether maternal vitiligo was associated with autoimmune, atopic, or neurodevelopmental disorders in offspring and whether associations differed by timing of maternal disease onset.

We used the Korean National Health Insurance Service and national birth registry databases to identify all live births from 2003 to 2016 (*n* = 5,069,152) and followed children through 2021 (Supplementary Fig 1, available via Mendeley at doi:10.17632/rxg2b32yn7.1). Maternal vitiligo was defined using ≥2 ICD-10 L80 claims within 1 year (positive predictive value, 91.2%). Onset was categorized as before pregnancy, during pregnancy, or after delivery. Offspring outcomes included autoimmune diseases (vitiligo, alopecia areata, psoriasis, and morphea), systemic autoimmune diseases (Graves’ disease, Hashimoto’s thyroiditis, and type 1 diabetes), atopic conditions (atopic dermatitis, urticaria, and asthma), and neuropsychiatric disorders (autism spectrum disorder, attention-deficit/hyperactivity disorder, and cerebral palsy). Covariates included maternal age, socioeconomic status, hypertension, diabetes, pregnancy complications, mode of delivery, and offspring characteristics (sex, birth year, and birth weight) (Table I).

**Table I:** Baseline characteristics of study population

Characteristics	Offspring born to mothers with vitiligo, no. (%) *n* = 2,049,088	Offspring born to mothers without vitiligo, no. (%) *n* = 20,064	Std diff
Mode of delivery (C/s)	1,860,036 (36.8)	7445 (37.1)	0.00554
Neonatal resuscitation	9323 (0.18)	31 (0.15)	0.00732
Labor induction	1,112,841 (22.0)	4309 (21.5)	0.01367
Maternal hypertension	60,559 (1.20)	234 (1.17)	0.00307
Maternal diabetes	264,815 (5.24)	1055 (5.26)	0.0006
Genitourinary infection during pregnancy	235,932 (4.67)	970 (4.83)	0.0076
Pre-eclampsia	56,603 (1.12)	215 (1.07)	0.00475
Eclampsia	5744 (0.11)	17 (0.08)	0.00922
Excessive weight gain during pregnancy	2467 (0.05)	0 (0.00)	0.00683
Socioeconomic status			0.096
Medicaid	41,164 (0.82)	116 (0.58)	
Q1	580,766 (11.5)	2107 (10.5)	
Q2	1,715,246 (33.97)	6248 (31.1)	
Q3	1,813,938 (35.93)	7420 (37.0)	
Q4	892,752 (17.7)	4148 (20.7)	
Missing	5222 (0.10)	25 (0.12)	
Sex-offspring (male)	2,600,136 (51.5)	10,178 (50.7)	0.01539
Birth weight			0.0074
Normal	4,829,150 (95.64)	19,177 (95.6)	
<1500g	7132 (0.14)	34 (0.17)	
1500-2500g	212,806 (4.21)	853 (4.25)	
Maternal age group			0.05545
<30 y	1,727,957 (34.2)	6360 (31.7)	
30-34 y	2,358,904 (46.7)	9629 (48.0)	
>35 y	962,227 (19.1)	4075 (20.3)	

Among 20,064 exposed and 5,049,088 unexposed offspring (median follow-up 9.8 years), maternal vitiligo was associated with higher risks of multiple immune-related diseases (Fig 1, Supplementary Fig 2, available via Mendeley at xxx). Offspring vitiligo risk was substantially elevated (adjusted hazard ratio [aHR] 7.05, 95% CI 6.53-7.62), with the highest risk when maternal vitiligo developed during pregnancy (aHR 9.88), followed by onset after delivery (7.34) and before pregnancy (6.55). Risks were also increased for alopecia areata (aHR 1.52) and psoriasis (aHR 1.89). Of systemic autoimmune diseases, only Graves’ disease was significantly elevated (aHR 2.02). Atopic conditions demonstrated modest risk increases—atopic dermatitis (aHR 1.19), urticaria (aHR 1.07), and asthma (aHR 1.05). No significant associations were observed for neuropsychiatric outcomes.

**Fig 1:**
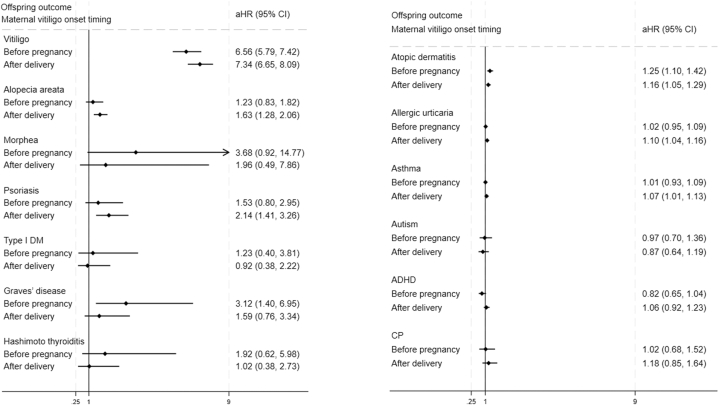
Autoimmune (with or without skin involvement), atopic, and neuropsychiatric outcomes in offspring born to mothers with vitiligo. *aHR*, Adjusted hazard ratio; *ADHD*, attention-deficit/ hyperactivity disorder; *CI*, confidence interval; *CP*, cerebral palsy; *type 1 DM*, type 1 diabetes mellitus.

In exploratory sex-stratified analyses, both male and female offspring had increased risks of vitiligo, alopecia areata, and atopic dermatitis. Elevated risks of psoriasis, morphea, and Graves’ disease were observed primarily among males. Although these conditions are often reported to be more common in females,^3^ our findings suggest sex-specific immunologic or hormonal modulation rather than differences in baseline prevalence.

The timing of maternal disease onset also influenced risk patterns. The highest risks when vitiligo was diagnosed during pregnancy support a potential role for intrauterine immune dysregulation or circulating autoantibodies.^4^^,^^5^ Elevated risks among offspring of mothers diagnosed after delivery suggest genetic susceptibility, whereas comparatively lower risks when vitiligo preceded pregnancy may reflect selection bias, whereby women with more severe or active disease may delay conception, resulting in a healthier prepregnancy subgroup.

This study has limitations. Claims-based diagnoses may introduce misclassification; residual confounding cannot be excluded; and surveillance bias may occur if children of mothers with chronic disease receive more medical attention. In addition, because multiple outcomes were evaluated, the possibility of type I error should be considered, as no formal correction for multiplicity was applied.

In conclusion, maternal vitiligo is associated with increased long-term risks of autoimmune and atopic diseases in offspring, reflecting potential combined genetic and intrauterine immune influences. These findings underscore the importance of maternal immune health during pregnancy and highlight the need for pediatric surveillance.

## Conflicts of interest

None disclosed.
